# Nuclear factor one B (*NFIB*) encodes a subtype-specific tumour suppressor in glioblastoma

**DOI:** 10.18632/oncotarget.8720

**Published:** 2016-04-13

**Authors:** Brett W. Stringer, Jens Bunt, Bryan W. Day, Guy Barry, Paul R. Jamieson, Kathleen S. Ensbey, Zara C. Bruce, Kate Goasdoué, Hélène Vidal, Sara Charmsaz, Fiona M. Smith, Leanne T. Cooper, Michael Piper, Andrew W. Boyd, Linda J. Richards

**Affiliations:** ^1^ Brain Cancer Research Unit, QIMR Berghofer Medical Research Institute, Brisbane, 4006, Queensland, Australia; ^2^ Leukaemia Foundation Research Laboratory, QIMR Berghofer Medical Research Institute, Brisbane, 4006, Queensland, Australia; ^3^ Queensland Brain Institute, The University of Queensland, Brisbane, 4072, Queensland, Australia; ^4^ School of Biomedical Sciences, The University of Queensland, Brisbane, 4072, Queensland, Australia; ^5^ Department of Medicine, The University of Queensland, Brisbane, 4072, Queensland, Australia

**Keywords:** glioblastoma (GBM), glioma, nuclear factor I B (NFIB), tumour suppressor gene, GBM subtype

## Abstract

Glioblastoma (GBM) is an essentially incurable and rapidly fatal cancer, with few markers predicting a favourable prognosis. Here we report that the transcription factor NFIB is associated with significantly improved survival in GBM. *NFIB* expression correlates inversely with astrocytoma grade and is lowest in mesenchymal GBM. Ectopic expression of NFIB in low-passage, patient-derived classical and mesenchymal subtype GBM cells inhibits tumourigenesis. Ectopic NFIB expression activated phospho-STAT3 signalling only in classical and mesenchymal GBM cells, suggesting a mechanism through which NFIB may exert its context-dependent tumour suppressor activity. Finally, NFIB expression can be induced in GBM cells by drug treatment with beneficial effects.

## INTRODUCTION

Glioblastoma (GBM; WHO grade IV astrocytoma) is the most common, and most lethal, primary malignant cancer of the central nervous system [1]. Despite surgery, radiotherapy and temozolomide chemotherapy, patients with GBM have a median survival of 14.6 months, with only 10.7% disease-free after two years [2]. Many genetic alterations and gene expression changes have been reported for GBM, yet those that contribute significantly to cellular transformation in this disease remain to be defined, as are informative molecular biomarkers that predict patient prognosis. Identification of these may inform new strategies to treat this almost uniformly fatal disease.

Histologically, GBM consists of proliferative, poorly differentiated cells of the glial lineage. While neural stem cells [3], oligodendrocyte precursor cells [4], astrocytes [3, 5] and neurons [5] all have been shown to be potential cells of origin of GBM in experimental models, the molecular mechanisms responsible for the lack or loss of differentiation in this disease are largely unknown. Nuclear factor one B (NFIB) is a phylogenetically conserved vertebrate transcription factor that promotes the differentiation of astrocytes from neural stem/progenitor cells during the process of gliogenesis in the developing mammalian central nervous system [6, 7]. Changes in the levels of NFIB protein directly increase astrogliogenesis-NFI transcription factors directly activate glial differentiation genes, such as glial fibrillary acid protein (*GFAP*) [8], myelin basic protein (*MBP*) [9] and fatty acid binding protein 7 (*FABP7*) [10]. In *Nfib* knockout mice, NFIB loss results in an increase in progenitor cells and a reduction and delay in astrocyte differentiation in the hippocampus and neocortex [6, 7, 11]. Conversely, ectopic overexpression of NFIB in the spinal cord induces precocious astrogliogenesis [12]. These studies suggest that NFI genes play a key role in the transition of proliferating progenitor cells to differentiated astrocytes. Indeed, loss of one allele of *NFIB* on chromosome 9p, leading to *NFIB* haploinsufficiency, occurs in 39% of GBM patients [13, 14]. Loss of 9p, which also includes the tumour suppressor *CDKN2A* besides *NFIB*, has been implicated directly in glioma progression [15]. Moreover, NFIB over-expression inhibits the transformation of chicken embryonic fibroblasts by nuclear oncogenes such as Myc and Jun [16]. Furthermore, *Nfib* was identified in insertional mutagenesis mouse screens designed to identify genes that, when mutated, increase the likelihood of developing GBM or other brain tumours [17–20]. Taken together, these observations underscore the possibility that as an inducer of astrocyte differentiation, NFIB could function as a tumour suppressor in astrocytic tumours. Here, we investigated the function of NFIB in human GBM and the potential clinical relevance of NFIB as a tumour suppressor in GBM biology.

## RESULTS

### *NFIB* expression correlates inversely with astrocytoma grade and is lowest in mesenchymal GBM

**Figure 1:**
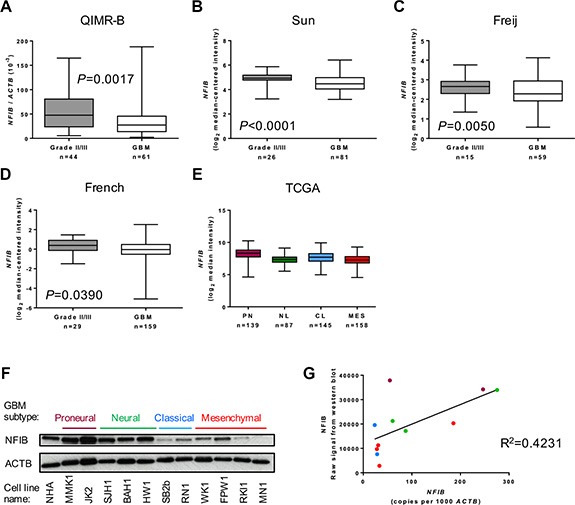
NFIB expression correlates inversely with astrocytoma grade (**A**) *NFIB* expression is higher in WHO grade II and III astrocytic gliomas (II/III) and lower in GBM. *NFIB* expression in patient tissue was determined by qPCR. *NFIB* expression also correlates inversely with astrocytic glioma grade in the independent (**B**) Sun [21], (**C**) Feije [22] and (**D**) French [23] microarray datasets. In the TCGA GBM dataset [26] (**E**) *NFIB* expression was highest in proneural GBM and lowest in mesenchymal GBM. *NFIB* expression in the proneural subtype is significantly different from all other subtypes (ANOVA *P* < 0.0001). (**F**) NFIB protein expression was highest in proneural and neural low-passage, patient-derived GBM cell lines and lowest in classical and mesenchymal lines as determined by immunoblot and (**G**) correlated with mRNA expression as determined by qPCR. Colour of dots corresponds to GBM subtypes in F.

We first investigated whether the level of *NFIB* expression corresponds to the degree of differentiation, or grade, of astrocytoma. We performed qPCR analysis of WHO grade II/III astrocytomas and oligoastrocytomas as well as WHO grade IV GBM. Compared to grade II/III tumours, we found that *NFIB* expression was significantly lower in GBM samples (Figure 1A). To verify this finding, we analysed the expression of *NFIB* mRNA in three independent larger cohorts for which expression profiling was available [21–23]. In all three datasets *NFIB* expression inversely correlated with astrocytoma grade (Figure 1B–1D).

GBM is diagnosed using histological criteria. However, mRNA expression profiling of individual GBMs has identified patterns of gene expression that have been used to define distinct molecular subtypes of GBM [24, 25]. The most widely used molecular classification system describes proneural, neural, classical and mesenchymal subtypes of GBM [25]. We therefore analysed whether *NFIB* expression correlated with these subtypes using data from The Cancer Genome Atlas (TCGA) dataset of patient tumour tissue, classified as either proneural, neural, classical or mesenchymal GBM [25–27]. We found *NFIB* expression was lowest in mesenchymal GBM and highest in proneural GBM (Figure 1E). To determine the level of NFIB protein expressed in GBM, we performed immunoblot analysis of GBM patient-derived cells lines (free of brain and stromal tissue that might express NFIB). This revealed that NFIB protein expression was reduced in patient-derived cell lines established from classical and mesenchymal GBM compared to those established from proneural and neural subtype GBM (Figure 1F).

We also investigated the correlation between *NFIB* mRNA and protein expression by comparing patient-derived GBM cell line qPCR data with densitometric measurement of our immunoblot results. This confirmed a direct correlation between *NFIB* mRNA and protein expression in GBM (Figure 1G). Collectively, these findings demonstrate that*NFIB* expression correlates inversely with astrocytoma grade and is lowest in mesenchymal GBM.

### *NFIB* expression and copy number correlates with glioma patient survival

**Figure 2:**
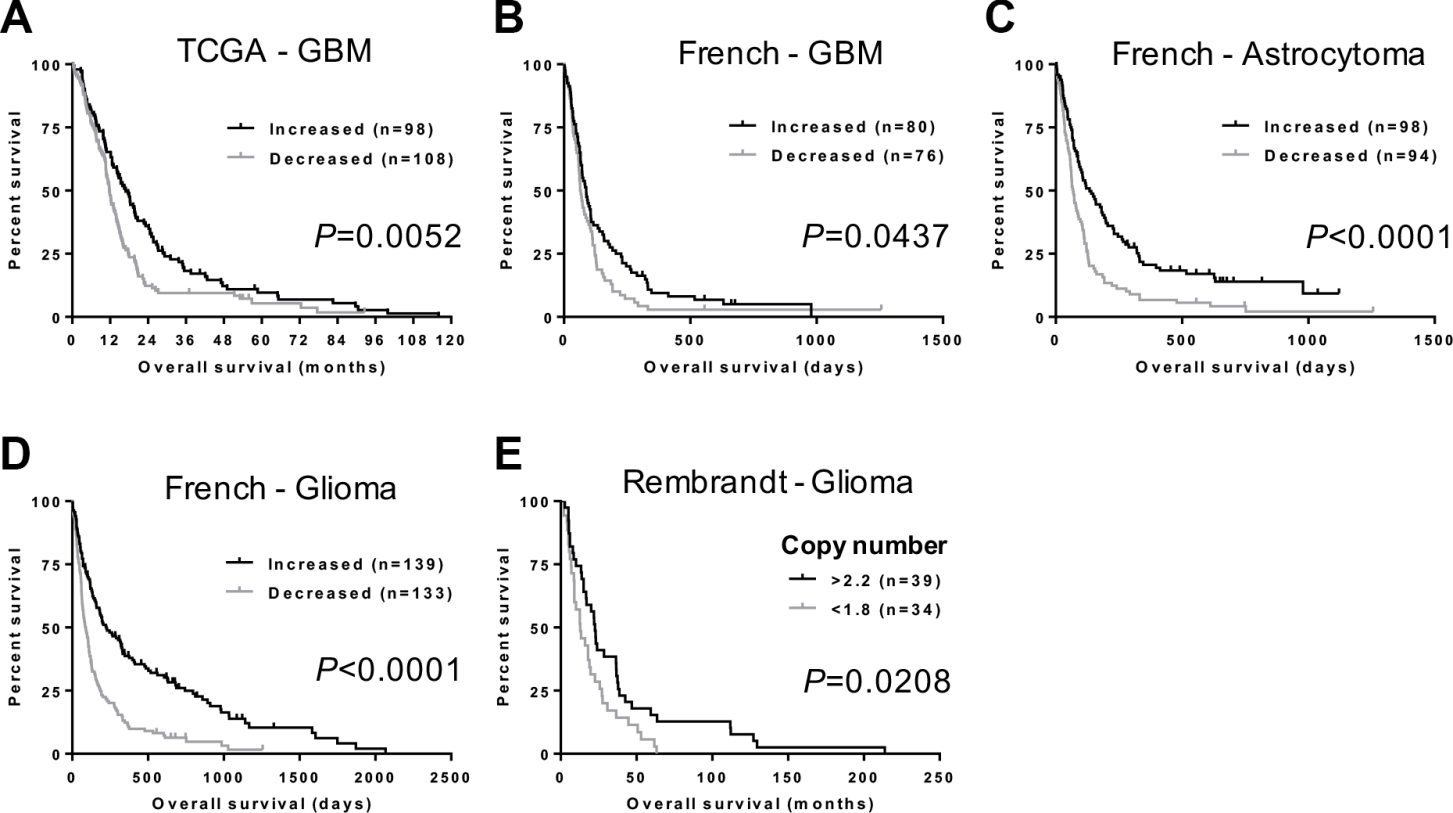
*NFIB* expression and copy number correlate with glioma patient survival (**A**) GBM patients with higher *NFIB* expression survive significantly longer than those with lower *NFIB* expression. Higher and lower are relative to mean *NFIB* expression for the entire cohort. Data are from the TCGA GBM dataset [27]. Increased *NFIB* expression also correlates with better survival for (**B**) GBM, (**C**) astrocytoma and (**D**) glioma in the French dataset [23]. (**E**) Glioma patient survival also worsens with reduced *NFIB* copy number in the Rembrandt glioma dataset [28]. Copy number values are the dataset analysis default settings.

We next investigated whether there was an association between *NFIB* expression and GBM patient survival. Using TCGA gene expression and patient survival data [26, 27], we found that GBM patients with higher *NFIB* expression survived significantly longer than those with lower *NFIB* expression (Figure 2A). Furthermore, using the independent French dataset [23], we found that the significant survival benefit associated with higher expression of *NFIB* existed for patients with GBM, astrocytoma and glioma (Figure 2B–2D). In addition, using the independent Rembrandt glioma dataset [28], we found that improved patient survival was also evident when *NFIB* gene copy number was considered independently of gene expression (Figure 2E). Thus, expression of *NFIB* is a prognostic factor that predicts improved survival for GBM, astrocytoma and glioma.

### Ectopic expression of NFIB in human classical and mesenchymal GBM inhibits tumour growth

**Figure 3:**
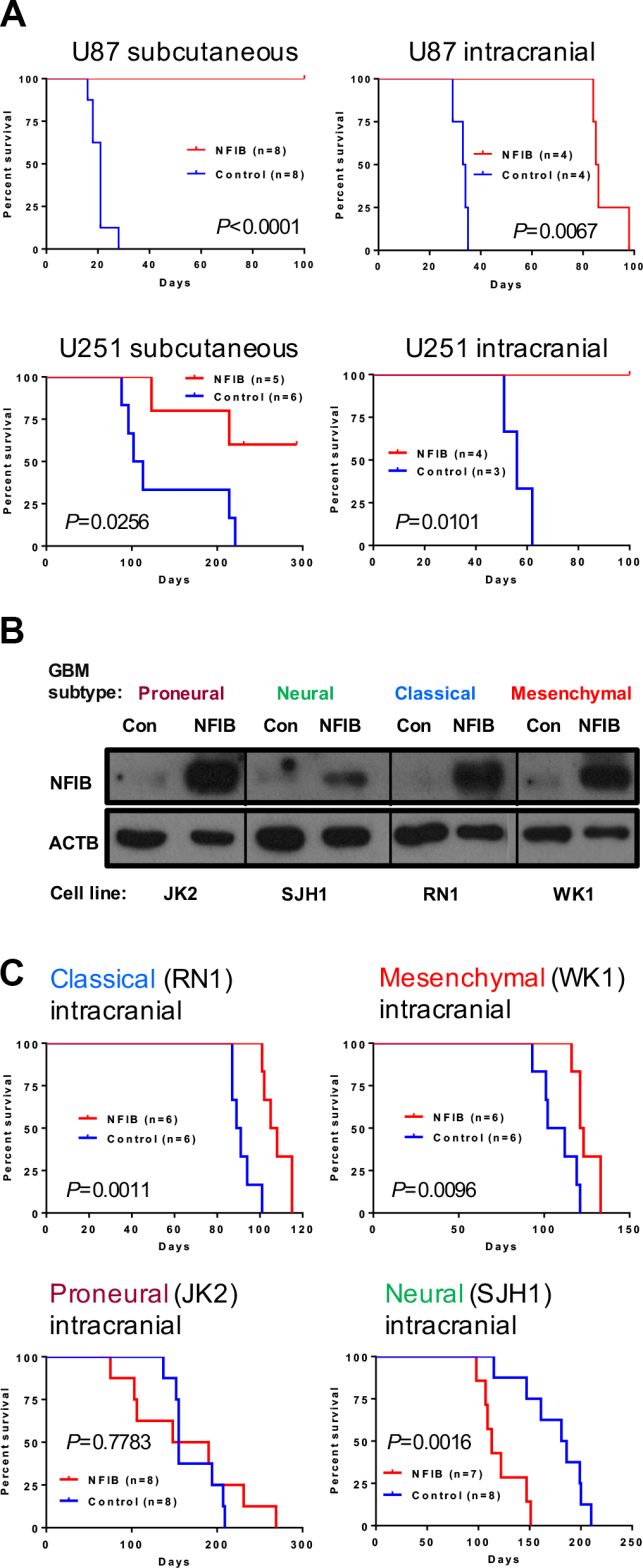
Ectopic expression of NFIB in human classical and mesenchymal GBM inhibits tumour growth (**A**) Both subcutaneous and intracranial xenograft tumour formation by (mesenchymal) U87 and U251 GBM cells expressing NFIB was significantly slower than vector control cells. (**B**) Western blot showing NFIB expression in patient-derived proneural, neural, classical and mesenchymal GBM cells lines expressing NFIB from the ubiquitin C promoter. (**C**) NFIB expression inhibited intracranial xenograft tumour formation by classical and mesenchymal GBM cells but not proneural or neural GBM cells.

To investigate whether increased expression of NFIB improves survival in GBM, we next examined the effect of ectopic expression of NFIB on tumour growth using two widely used models of human GBM, U87 and U251 cell lines, which express low levels of NFIB compared to normal human brain. When both cell lines were transfected with *Nfib* and injected either subcutaneously or intracranially they formed xenograft tumours significantly more slowly, or not at all, in NOD/SCID mice (Figure 3A) compared to xenografts of control transfected cells. These two cell lines have a phenotype closest to mesenchymal GBM [25] and therefore provided initial evidence that NFIB could suppress tumour formation in this aggressive tumour subtype. We also repeated this experiment in primary GBM cell lines of low passage, established from patients with proneural, neural, classical or mesenchymal subtype GBM [29–32]. Four patient-derived GBM cell lines (one of each molecular subtype) were transduced with a lentivirus expressing either *Nfib* from the ubiquitin C promoter or the same vector without the *Nfib* coding sequence (Figure 3B). Ectopic expression of NFIB inhibited the intracranial xenograft tumour growth of both classical and mesenchymal GBM (Figure 3C) but did not inhibit the growth of proneural or neural GBM. Indeed, NFIB expression appeared to enhance the growth of the neural GBM subtype, behaving more like an oncogene. These data suggest an interesting and important context-dependent role for NFIB in GBM xenograft growth.

### Ectopic expression of NFIB induces differentiation, and inhibits proliferation and self-renewal of human mesenchymal and classical GBM subtypes

**Figure 4:**
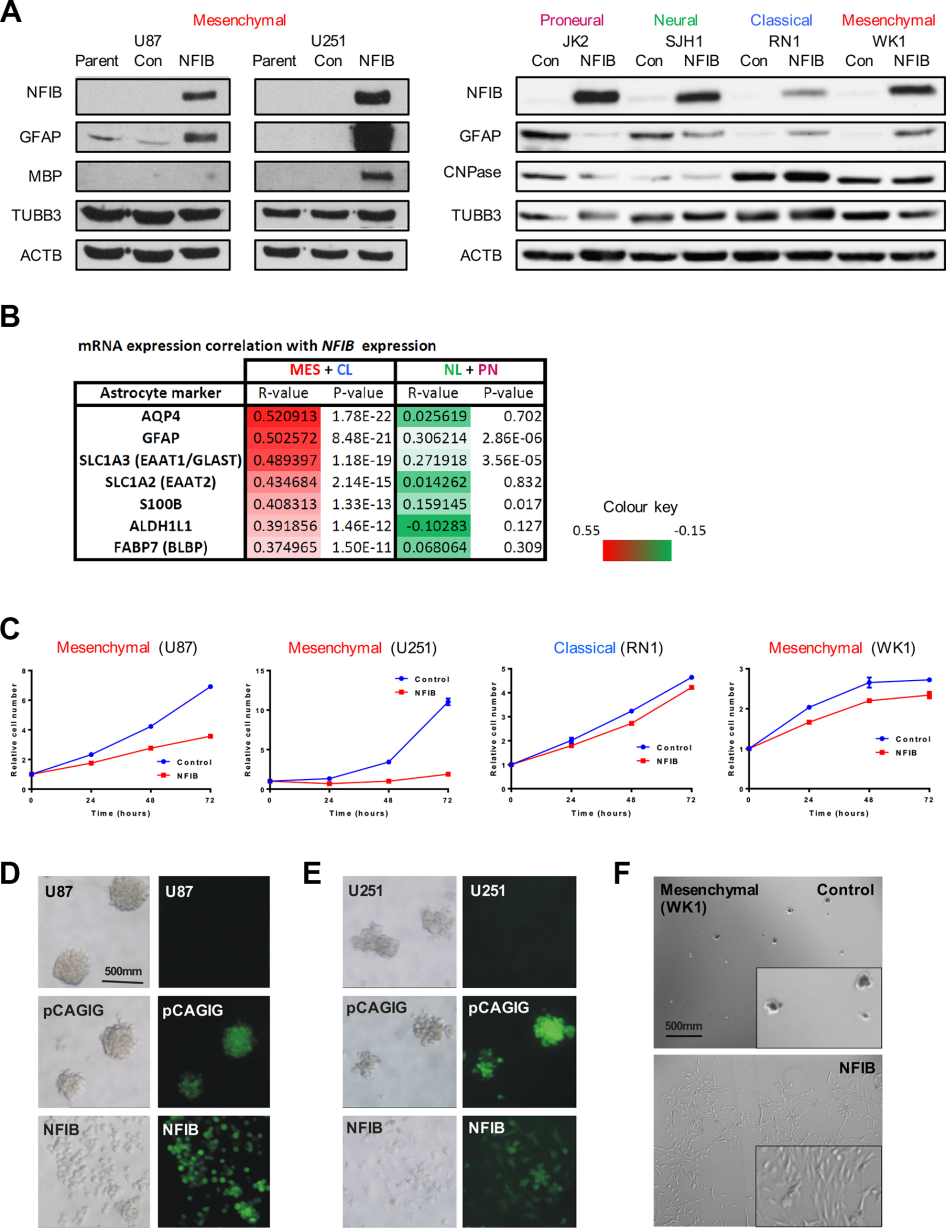
Ectopic expression of NFIB in human mesenchymal GBM induces differentiation, inhibits proliferation and inhibits self-renewal (**A**) Expression of the astrocytic marker GFAP was observed in mesenchymal (U87, U251, WK1) and classical (RN1) GBM cells in response to NFIB expression, but not in proneural (JK2) or neural (SJH1) GBM cells. Changes in expression of the oligodendrocyte markers MBP or CNPase were less pronounced. (**B**) NFIB expression correlates with expression of astrocyte-associated genes in classical and mesenchymal GBM but not proneural and neural GBM in the TCGA GBM gene expression dataset. Pearson correlation coefficient values are shown for individual GBM subtypes as well as combined CL+MES and PN+NL subtypes. (**C**) NFIB expression inhibited proliferation of mesenchymal and classical GBM cells as measured by MTS assay. NFIB expression inhibited tumoursphere formation in mesenchymal (**D**) U87, (**E**) U251 and (**F**) WK1 GBM cells only.

We next sought to identify possible mechanisms by which NFIB exerted its GBM subtype-specific tumour-suppressive effect. Based on astrogliogenic function of NFIB during brain development, we reasoned that NFIB might also up-regulate the expression of glial differentiation genes in GBM cells. In both established and patient-derived GBM cells, we observed an increase in expression of GFAP and, to a lesser extent, the oligodendrocytic markers MBP or CNPase, in response to ectopic NFIB expression in mesenchymal and classical GBM lines (Figure 4A). However, we saw a decrease in GFAP expression in the proneural and neural GBM cell lines, suggesting a lack of differentiation and supporting our previous observations in xenograft models where NFIB afforded no survival benefit and was even oncogenic in the neural subtype.

To determine if these relationships occur in primary tissues, we assessed the correlation of *NFIB* mRNA expression with astrocyte-associated markers [33, 34] in the TCGA GBM gene expression dataset [27]. This analysis revealed that astrocytic markers displayed a significant positive correlation with *NFIB* mRNA expression in the mesenchymal and classical tumours but not the proneural and neural tumour subtypes (Figure 4B). These results, together with our GBM cell line models, provide evidence that NFIB is a potent regulator of glial differentiation in GBM.

In the developing brain, NFIB has been shown to regulate proliferation via the Notch/Hes pathway [35], thus allowing cells to undergo differentiation. We therefore investigated whether over-expression of NFIB could reduce GBM cell proliferation. Consistent with the xenograft survival data, we observed a reduction in cell proliferation in response to NFIB expression in both established and patient-derived mesenchymal (U87, U251 and WK1) and classical (RN1) GBM cell lines (Figure 4C). This effect, however, was not evident for the other two GBM subtypes (Supplementary Figure 1A).

Tumourigenicity is often paralleled by the ability of GBM cells to form tumourspheres [36–39], an assay of the capacity of cells to self-renew. We therefore investigated the effect of NFIB expression on tumoursphere formation by GBM cell lines. U87, U251 and WK1 cells were readily passaged as tumourspheres when transfected with control plasmids. However when expressing NFIB, they failed to form tumourspheres and instead grew as adherent cultures, similar to growth in serum-containing medium (Figure 4D–4F). In contrast, tumoursphere growth of the proneural, and classical GBM lines was not altered by NFIB expression and growth of the neural line was enhanced (Supplementary Figure 1B).

### NFIB activates phospho-STAT3 signalling in classical and mesenchymal GBM cells

**Figure 5:**
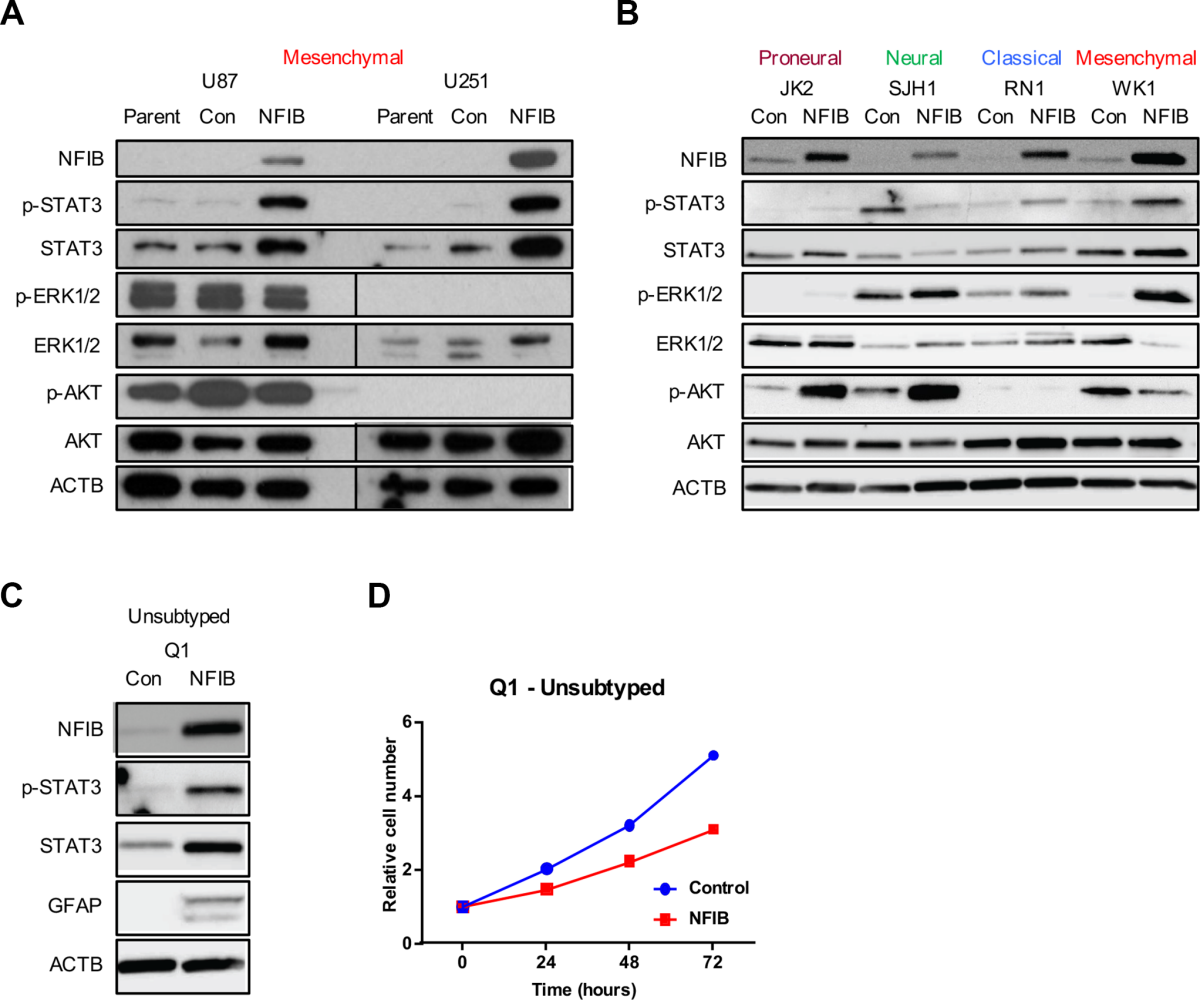
STAT3 signalling predicts NFIB function in GBM cell lines Changes in p-STAT3 but not ERK or AKT signalling paralleled the activity of NFIB in proneural, neural, classical and mesenchymal GBM cells - increased STAT3 phosphorylation was observed in (**A)** U87 and U251 (mesenchymal) GBM cells and in (**B)** classical and mesenchymal patient-derived GBM lines in response to increased NFIB expression. No change in p-STAT3 was observed in proneural cells and reduced expression was seen in neural GBM cells. In contrast no consistent correlation was observed between NFIB activity and either ERK or AKT signalling. (**C**) Expression of NFIB in the low-passage, unsubtyped GBM cell line Q1 was associated with increased expression of p-STAT3, increased expression of the astrocyte marker GFAP and (**D**) inhibition of cell proliferation.

Given the subtype-specific effects of NFIB in GBM, we sought to identify additional determinants of NFIB function. Phospho-STAT3 (p-STAT3) has been shown to bind to the GFAP promoter and activate its transcription [40], and is required for astrocyte differentiation [41]. We therefore investigated p-STAT3 signalling in proneural, neural, classical and mesenchymal GBM cells in response to increased NFIB expression. Paralleling the GBM subtype-specific effects of NFIB, we observed increased p-STAT3 in classical and mesenchymal GBM cells, no change in p-STAT3 expression in proneural GBM cells and a decrease in p-STAT3 in neural GBM cells (Figure 5A and 5B).

As a control we also investigated ERK and AKT signalling in proneural, neural, classical and mesenchymal GBM cells as both signalling pathways are frequently activated in GBM [26, 42]. In contrast to the changes in p-STAT3 levels that paralleled phenotypic responsiveness to NFIB, inconsistent changes in both ERK and AKT signalling pathways were observed in response to increased NFIB (Figure 5A and 5B). Increased ERK1/2 phosphorylation was seen in proneural, neural, classical and mesenchymal low-passage GBM cells, but no changes in U87 and U251 cells. Increased AKT phosphorylation was evident in proneural and neural GBM lines and reduced AKT phosphorylation was observed in one of the mesenchymal lines, with no change in the others. While changes in both signalling pathways occurred in response to increased NFIB expression, their failure to parallel the phenotypic changes observed in the corresponding GBM cell subtypes suggests that neither pathway has as significant a role as p-STAT3 in determining NFIB-responsiveness.

These results suggest that p-STAT3 may mediate NFIB-responsiveness in GBM and could act as a biomarker of beneficial effect. To investigate this, we selected an unsubtyped low-passage GBM cell line (Q1) and ectopically expressed NFIB. Immunoblotting revealed increased p-STAT3 in these cells in response to NFIB (Figure 5C). Consistent with this observation, we also observed increased GFAP expression (Figure 5C) and reduced cell proliferation (Figure 5D) in response to increased NFIB expression. Exome sequencing showed that this cell line was *CDKN2A* null and expressed EGFRvIII, mutations most often associated with classical GBM [25]. Collectively these data suggest that pSTAT3 signalling may be a determinant of NFIB function in GBM.

### NFIB expression can be induced by drug treatment of GBM cells and promote beneficial effects

**Figure 6:**
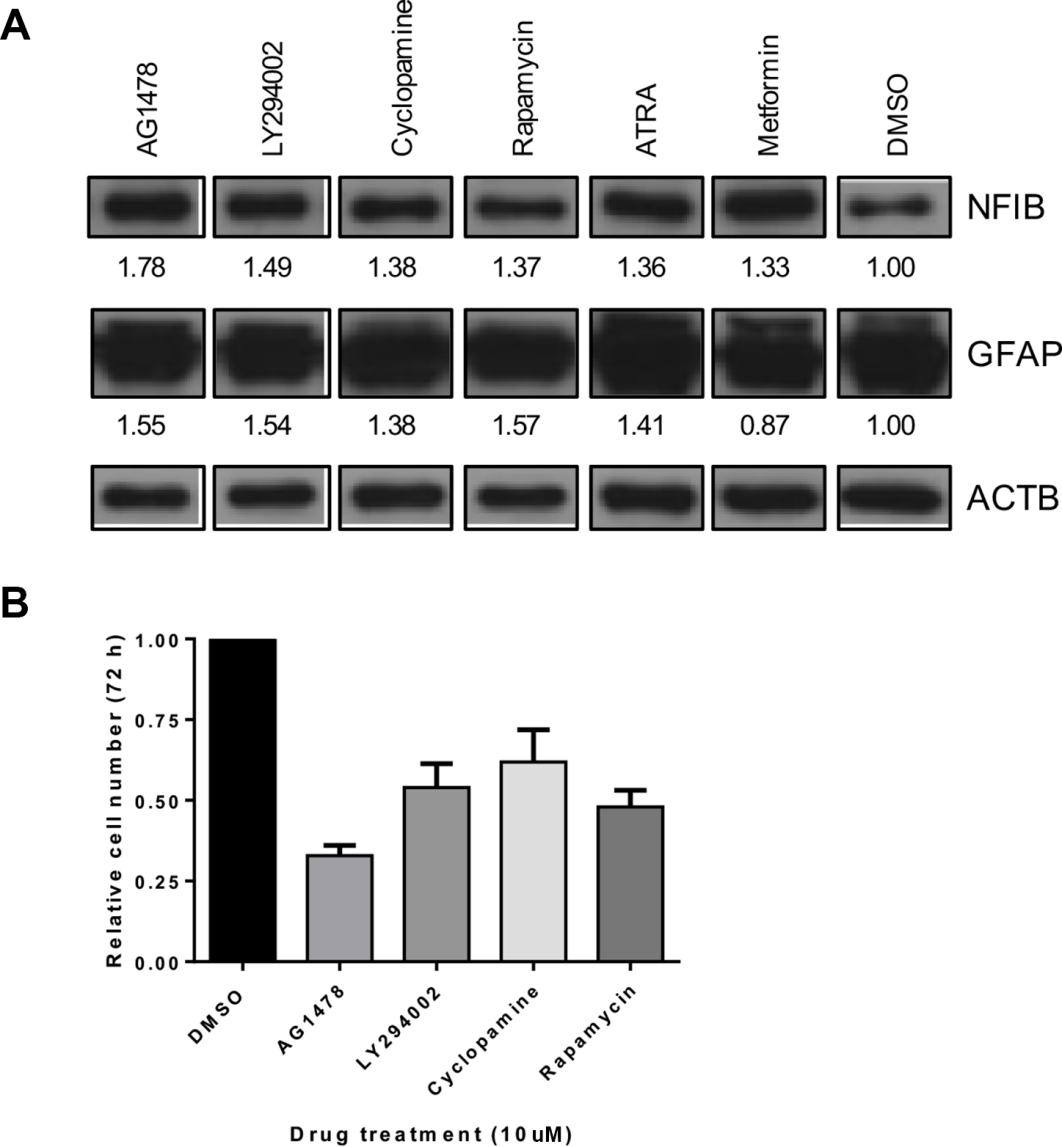
NFIB expression in GBM cells can be increased by drug treatment and is associated with reduced proliferation (**A**) A small pilot study identified six drugs (at 10 μM concentration) that increased NFIB expression in Q1 cells by > 30% (also see Supplementary Figure 2). Five of these drugs also increased GFAP expression by > 30%. Numbers below each panel represent the fold-change in western blot signal intensity, measured by densitometry, relative to DMSO-treated control cells and normalised to β-actin expression. (**B**) Four of these drugs were found to inhibit Q1 proliferation by 38–67%.

Because *NFIB* is rarely mutated in glioma and at least one copy of the gene is present in over 95% of GBM [27, 43], we investigated whether NFIB expression could be increased by drug treatment of GBM cells. As exome sequencing showed that Q1 cells possessed two wild type *NFIB* alleles, (data we did not have available for the other cell lines we used) we used this line for these experiments. Q1 cells were treated with 31 different drugs, each at 10 μM, for 72 hours (Supplementary Figure 2). NFIB expression was investigated by western blotting, quantified by densitometry and normalised to β-actin expression for comparison with vehicle-treated control cells. Six drugs were found to increase NFIB expression by more than 30% (Figure 6A). To determine whether increased NFIB expression was associated with evidence of differentiation, we also investigated GFAP expression by western blotting (Figure 6A). Five of the six drugs were found to also increase GFAP expression by more than 30%. Finally, the top four drugs were investigated for their effect on cell proliferation (Figure 6B). All were found to inhibit cell proliferation by between 38 and 67%, with the most effective drug being AG1478, an epidermal growth factor receptor inhibitor. These findings demonstrate that NFIB expression can be induced in GBM cells by drug treatment, at levels associated with tumour-suppressive effects.

## DISCUSSION

Here we report that *NFIB* expression correlates inversely with astrocytoma grade. In GBM, *NFIB* expression is lowest in mesenchymal GBM, highest in proneural GBM and is associated with significantly improved survival in specific subtypes of this disease. Importantly, ectopic expression of NFIB inhibits cell proliferation and tumourigenesis in patient-derived cell line and xenograft models of classical and mesenchymal GBM, to a degree determined by the level of NFIB expression, but not proneural and neural subtype GBM. The expression of astrocyte-associated markers is up-regulated in three mesenchymal and two classical GBM cell lines in response to NFIB expression, which also occurs in primary tumours of these subtypes. Interestingly, pSTAT3 is increased only in GBM cells where ectopic NFIB expression promotes differentiation. Thus, pSTAT3 signalling may be required for NFIB to induce differentiation and inhibit tumourigenesis in GBM. These results demonstrate that NFIB is a positive prognostic factor for this disease and a tumour suppressor in a specific subset of GBM.

A surprising finding from our study was the oncogenic effect of NFIB over-expression in neural GBM. While reduced expression or inactivation of other tumour suppressor genes, including *PTEN*, *NF1*, *ATRX* and *DAXX*, has been associated with specific GBM subtypes, we are not aware of any other wild type gene that behaves as a tumour suppressor in some GBM subtypes and as an oncogene in others. In addition, the subtype-specific context of NFIB function may indicate potential mechanisms by which this gene functions in GBM. As NFIB expression in proneural and neural subtype GBM is not reduced, genetic/epigenetic alterations may have rendered these subtypes insensitive to the action of NFIB. This is consistent with the neural pattern of gene expression observed in these two subtypes and may explain their resistance to the pro-astrocytic effects of increasing NFIB. In contrast, the lower level of NFIB in classical and mesenchymal GBM may be due to genetic/epigenetic changes that reduce NFIB expression in these two subtypes, resulting in their poorly-differentiated phenotype yet leaving them sensitive to the effects of re-expressed NFIB. Interestingly, the oncogenic effect of NFIB in neural GBM is akin to the action of NFIA in maintaining the glial progenitor cell pool in the embryonic spinal cord [12] and perhaps analogously promotes the propagation of the glioma stem cell compartment in this subtype. These results demonstrate that targeted treatment of GBM may need to be considered in conjunction with GBM subtype/genotype information to ensure not only effective treatment choice, but to avoid treatments which may exacerbate tumour growth.

In addition to glioma, NFIB has been linked with several other cancers, in either an oncogenic or tumour suppressive context. *MYB*-*NFIB* and *MYBL1*-*NFIB* gene fusions frequently occur in adenoid cystic carcinoma [44–47]. NFIB is likely to have a tumour suppressive role independent of MYB(L) in this cancer as truncation mutations specific to NFIB have also been found as have translocations involving other partners (e.g. *NFIB-MAN1A1*, *NFIB-PTPRD*, *NFIB-NKAIN2*, *NFIB-XRCC4* and *NFIB-AIG1*). *HMG2A-NFIB* fusions have been reported in lipomas while other tumour-associated translocations involving NFIB include *MPDZ-NFIB*, *NFIB-FREM*, *NFIB-HEATR5B*, *NFIB-STRN* and *NFIB-ZDHHC21*. Increased copy number and expression of *NFIB* has been reported for small cell lung cancer [48], prostate cancer [49] and triple negative breast cancer [50] consistent with an oncogenic role in these cancers. Conversely, NFIB has a tumour suppressor role in medulloblastoma, osteosarcoma [51] and cutaneous squamous cell carcinoma [52].

*NFIB* is one of four genes that comprise the Nuclear Factor One family of transcription factors (*NFIA*, *NFIB*, *NFIC* and *NFIX*) [53, 54]. Like NFIB, NFIA [55, 56] and NFIX [57] also have been implicated in glioma. Interestingly, NFIA also can behave as either a tumour suppressor or an oncogene in glioma. Three studies provide evidence that NFIA can act as a tumour suppressor: 1) increased expression of NFIA is observed in lower grade astrocytomas and is associated with improved survival [58], 2) NFIA inhibits the oncogenic transformation of chicken fibroblasts when overexpressed [16] and 3) inactivation of NFIA by transposon insertion potentiates tumour formation in the mouse brain [17–20]. As an oncogene, NFIA has been reported to promote tumourigenesis in an EGFR-vIII; SV40-LargeT-transformed neural stem cell model of GBM, as well as driving neural stem cell proliferation and producing a less differentiated phenotype [55]. Thus both NFIA and NFIB appear to function in a genetic context-dependent manner as either tumour suppressors or oncogenes.

While recent studies are helping define the genetic events that give rise to GBM, less is known about the genetic changes that accompany the progression of low-grade to high-grade glioma. Our observation that NFIB expression decreases with increasing glioma grade, together with the demonstration of its tumour-suppressive effects in GBM, suggest that NFIB loss may be a contributory factor in glioma progression. This hypothesis is supported by a genome-wide study of genetic alterations associated with grade II and III gliomas revealing loss of heterozygosity of *NFIB* with increasing glioma grade [43]. Similarly, while 90% of GBM arises *de novo*, there is evidence that proneural GBM progresses over time to mesenchymal GBM [59]. Our findings suggest that loss of NFIB expression may be a contributing factor in this process.

Although the molecular mechanisms by which NFIB mediates its anti-tumour effects in GBM remain to be defined, our findings suggest a role for p-STAT3. STAT3 signalling is detected in many cancers [60, 61], including GBM [62, 63–65] and may contribute to GBM pathogenesis by promoting cell proliferation and survival, immune suppression, invasion, and angiogenesis. Recent studies in GBM and other tumours, however, have suggested an alternative role for STAT3 in tumour suppression (reviewed in [66, 67]). STAT3, for example, has been demonstrated to function as a tumour suppressor in GBMs that have lost PTEN expression [68]. STAT3 is also both necessary and sufficient for astrocyte differentiation, a function dependent upon phosphorylation of Tyr-705 [41]. STAT3, like NFIB, also binds the promoter of GFAP and activates GFAP transcription during astrocytic differentiation of neural progenitor cells [40]. Interestingly, NFIA facilitates STAT3 binding to the GFAP promoter through demethylation of the cognate STAT binding site [69], a function that may extend to NFIB. An explanation for these divergent outcomes in response to STAT3 signalling is suggested by an observation by Carro *et al.* [70]. This study found that STAT3 signalling alone was insufficient to transform neural stem cells. However, malignant transformation to mesenchymal subtype GBM resulted when STAT3 signalling occurred in combination with C/EBP signalling. STAT3 signalling therefore may facilitate different phenotypic outcomes determined by additional signalling input or interaction with other transcription factors. Identification of gene promoters bound by both STAT3 and NFIB may help define key downstream mediators of the response to combined STAT3 and NFIB signalling.

Finally, as NFIB is expressed by GBM cells including GBM cancer stem/tumour-initiating cells [71], and is rarely homozygously deleted or mutated [27], it may have therapeutic benefit in GBM if its expression can be increased. Our pilot drug screen confirmed this, providing proof-of-principle that induction of NFIB expression in GBM cells is a potential therapeutic strategy that warrants further investigation.

Since a defined subset of GBM retains responsiveness to glial differentiation factors such as NFIB has important implications for the management of this cancer, as the ability to induce a more differentiated phenotype is a potential adjunctive therapy for GBM. Identification of blood-brain barrier permeable drugs that induce NFIB expression in GBM cells, together with the identification of the GBM genotypes in which NFIB has a tumour-suppressive effect, may define a novel therapeutic strategy to augment the management of this almost uniformly fatal disease [72].

## MATERIALS AND METHODS

### Tumour tissue and cell culture

Patient tumour tissue was collected following informed consent and with human ethics approval from the QIMR Berghofer Medical Research Institute and Royal Brisbane and Women's Hospital human research ethics committees. All human studies have been performed in accordance with the ethical standards laid down in the 2013 version of the 1964 Declaration of Helsinki. Tumour tissue was examined by a neuropathologist to determine tumour type and grade. Patient-derived cell lines [30–32, 73–76] were established as reported previously [29]. These were cultured as adherent monolayers in matrigel (BD Biosciences)-coated vessels using RHB-A stem cell culture medium (StemCells Inc) supplemented with 20 ng/ml EGF (Gibco) and 10 ng/ml FGFb (Gibco) or as tumourspheres using StemPro NSC SFM (Invitrogen). U87 and U251 cells (obtained from The University of Queensland and authenticated by PCR-based short tandem repeat profiling by the QIMR-B DNA Sequencing and Genotyping facility within the past 12 months) were maintained in RPMI160 medium (Gibco) supplemented with 10% foetal bovine serum (Gibco), 2 mM L-glutamine, 25 mM HEPES, 25 mM sodium bicarbonate, 100 U/ml penicillin and 100 μg/ml streptomycin. Cells were cultured in 5% CO_2_ / 95% humidified air at 37°C. GBM subtyping was performed as described [25].

### Cell transfection

N-terminal, HA-tagged *Nfib* was cloned into the expression vector pCAGIG (in which GFP is translated from the same mRNA as *Nfib*) and the lentiviral expression vector pF ubc MCS IRES mCherry hygro, a derivative of pF GVP hygro. U87 and U251 cells were transfected with pCAGIG HA-*Nfib* or empty vector and selected for stable expression of NFIB by three rounds of FACS for GFP. Patient-derived GBM cell lines were transduced with lentivirus produced from HEK293FT cells transfected with pF ubc HA-*Nfib* IRES mCherry hygro (or empty vector), pVSV-G and psPAX2, and selected by hygromycin resistance. Lentiviral transduction was performed by adding 1 ml of unconcentrated lentivirus to adherent cells in 4 ml of medium in a 6-well tissue culture plate with a final concentration of polybrene of 1 μg/ml followed by centrifugation at room temperature for 45 minutes at 440 g.

### RNA, cDNA and qPCR

RNA was extracted from adherent cell cultures using TRIzol (Invitrogen), as recommended by the manufacturer. cDNA was prepared with SuperScript III (Life Technologies) from 2 μg of DNA-free total RNA, using random hexamers (NEB Biolabs), and RNaseH (NEB Biolabs) digestion to remove residual RNA. Quantitative polymerase chain reaction (qPCR) was performed using SYBR green PCR master mix (Invitrogen), 2 μM of gene-specific primers (Sigma Aldrich), 50 ng of cDNA and a Rotor-Gene 3000 (Corbett Life Science). Reactions were incubated for 10 minutes at 95°C followed by 40 cycles of 20 seconds at 95°C, 30 seconds at 55°C, and 40 seconds at 72°C. Results were normalised to *ACTB*. Gene-specific qPCR primers were *NFIB* (F899 5′-CACATTGCACAAACCCAGCA-3′ and R1016 5′-CTTCCTGATTGTCCAGAATCTT-3′) and *ACTB* (F561 5′-CACACTGTGCCCATCTACGA-3′ and R688 5′-GTGGTGGTGAAGCTGTAGCC-3′).

### Immunoblotting

Immunoblotting was performed with 30–50 μg of total cell lysate, separated by 10% SDS-PAGE and transferred to PVDF membrane (BioRad). Protein lysates were prepared using Martin's lysis buffer (25 mM TrisHCl pH 8, 150 mM NaCl, 1% Triton X-100, 1 mM sodium vanadate, 1 mM sodium fluoride and 1x protease inhibitors (cOmplete ULTRA, Roche)). PVDF membranes were blocked with 5% low fat skim milk powder (Diploma) in PBS-T. Primary antibodies used were NFIB (Abcam, ab11989, 1:1000), GFAP (Dako, 1:1000), MBP (Sigma, 1:750), CNPase (Abcam, 1:1000), TUBB3 (Promega, 1:1000), ERK (Cell Signaling, 1:1000), phospho-ERK (Cell Signaling, 1:1000), AKT (Cell Signaling, 1:1000), phospho-AKT (Ser-473) (Cell Signaling, 1:1000), STAT3 (Cell Signaling, 1:1000), phospho-STAT3 (Tyr-705) (Cell Signaling, 1:1000) and ACTB (Sigma, 1:5000); secondary antibodies were anti-rabbit IgG-HRP (Dako, 1:3000) and anti-mouse IgG-HRP (Dako, 1:3000). Protein concentration was quantified by Bradford assay.

### GBM xenograft models

GBM xenograft models were initiated in 5-week old female NOD/SCID mice housed under pathogen-free conditions. For intracranial cell injections, 200,000 cells in 2 μl of 10 ng/ml laminin in PBS were injected 3 mm below the brain surface, 1.6 mm rostral of the bregma and 0.8 mm right of the midline, using a 25G Hamilton needle and 2 μl syringe in a stereotaxic frame. Mice were anaesthetised with 2% isoflurane (Abbott) in oxygen at a flow rate of 0.8 litres per minute and given 100 μg of carprofen (Pfizer) subcutaneously for analgesia. The burr hole through which the cells were injected was sealed with bone wax and the midline scalp incision was closed with Vetbond (3M) tissue adhesive. Subcutaneous cell injection was performed with 500,000 cells in 100 μl PBS, injected into the right flank with a 21G needle and 1 ml syringe. Mice were euthanased when they exhibited signs of significant morbidity (hunching, weight loss, rough coat, ataxia, head tilt, paralysis). All studies were conducted according to protocols approved by the Animal Ethics Committee of the QIMR Berghofer Medical Research Institute. The “Principles of laboratory animal care” (NIH publication No. 86–23, revised 1985) were followed as well as the “Australian code of practice for the care and use of animals for scientific purposes”, 8th edition 2013 and the “Queensland Animal Care and Protection Act 2001”.

### Proliferation assays

Proliferation assays were performed using MTS reagent (Promega) as recommended by the manufacturer. Assays were performed at least three times and representative results are shown.

### *In vitro* limiting dilution assays

*In vitro* limiting dilution assays were performed as previously described [77].

### Drug treatment

1.5 × 10^5^ cells were cultured as adherent monolayers, in matrigel-coated 12-well plates in serum-free RHB-A medium supplemented with 20 ng/ml EGF and 10 ng/ml FGFb. Three hours after plating, cells were treated with 10 μM drug for 72 hours. Drugs were dissolved in DMSO at a stock concentration of 50 mM, diluted with PBS to a working concentration of 200 μM and added to cells in culture at a dilution of 1:20.

### Microarray analysis of gene expression

TCGA gene expression data were analysed using cBioPortal [13, 14] or using log-transformed gene expression data from the UCSC Cancer Browser normalised by subtracting the mean value of each gene set. NFIB expression in the Sun [21], Freij [22], French [23] and Lee [78] datasets was investigated using ONCOMINE [79] using probeset 209289_at. NFIB expression data in the Rembrandt dataset used the same probeset and was analysed within the Rembrandt portal [28]. All glioma samples analysed contained an astrocytic component.

### Statistical analysis

Unless otherwise noted, data are presented as mean and standard error of the mean (SEM). Student's *t*-test (unpaired, two-tailed) was used to compare two groups of independent samples to determine the probability of difference and ANOVA to compare multiple independent groups. Correlation coefficients were determined using parametric linear regression analysis. All graphs were generated using GraphPad Prism 6. *P* < 0.05 was considered statistically significant.

## SUPPLEMENTARY MATERIALS FIGURES


