# Supplementary material for: Nuclear factor one B (NFIB) encodes a subtype-specific tumour suppressor in glioblastoma
Source: Oncotarget. 2016 Apr 13;7(20):29306–20. doi: 10.18632/oncotarget.8720 (PMC5045397; doi:10.18632/oncotarget.8720)
Supplement: Supplementary file 1 [file oncotarget-07-29306-s001.pdf]

Nuclear factor one B (*NFIB*) encodes a subtype-specific tumour suppressor in glioblastoma

Supplementary Materials

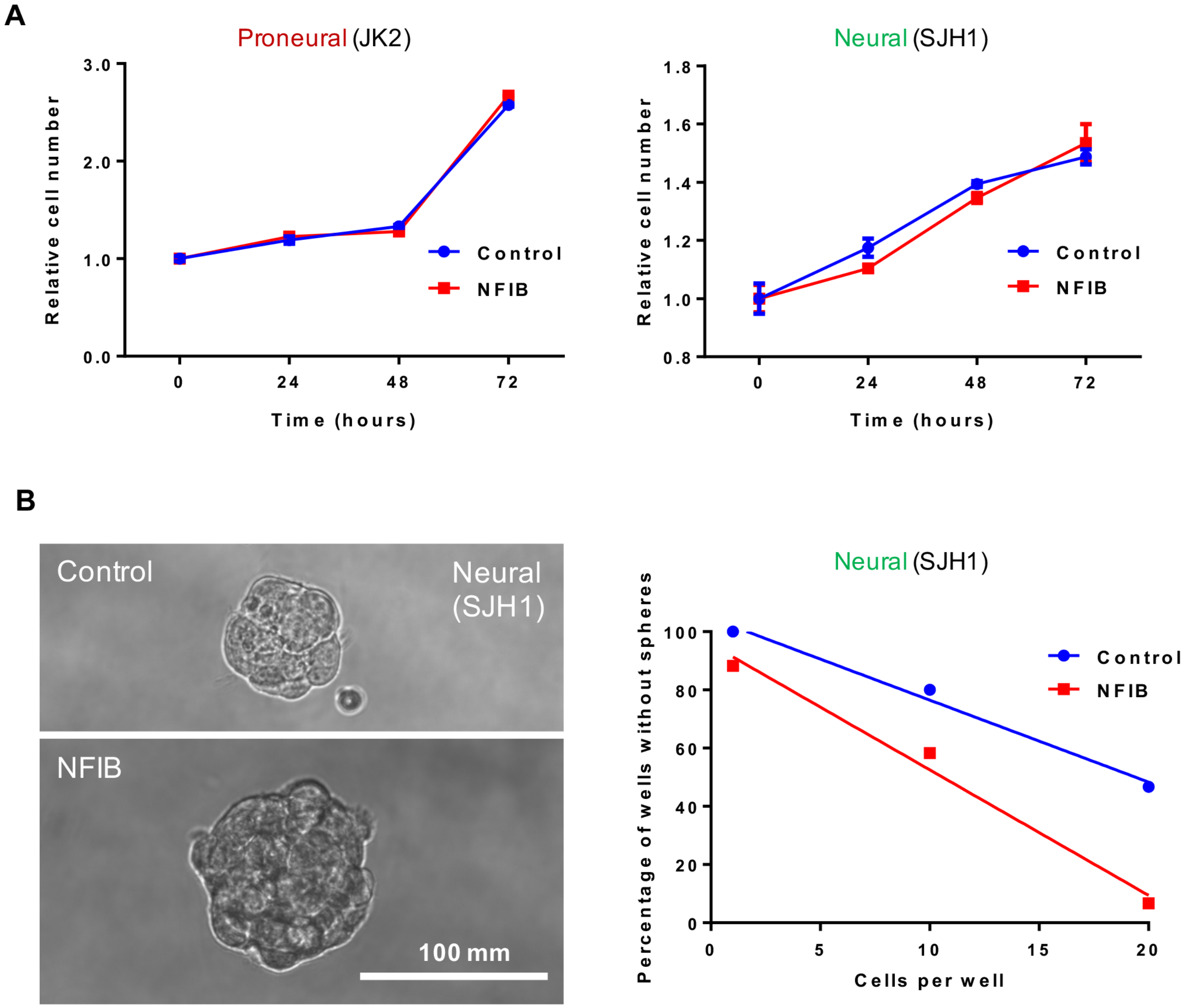

Supplementary Figure S1: Increased NFIB expression did not alter the proliferation of proneural or neural GBM cell lines although it enhanced tumoursphere formation by neural GBM cells. (**A**) Representative MTS assays for proneural (JK2) and neural (SJH1) GBM cell lines expressing NFIB from the ubiquitin C promoter. (**B**) Both tumoursphere growth and the frequency at which tumourspheres were initiated – both *in vitro* measures of tumourigenicity - were enhanced by increased expression of NFIB in neural (SJH1) GBM cells. Representative tumourspheres (left) and *in vitro* limiting dilution assay (right) for neural (SJH1) GBM cells expressing NFIB from the ubiquitin C promoter versus vector-control cells. Cells were seeded in 96-well plates at 1, 10 or 20 cells per well, in 100 µl of serum-free RHB-A medium supplemented with 20 ng/ml EGF and 10 ng/ml FGFb, and tumoursphere growth was recorded after 7 days of culture at 37°C.

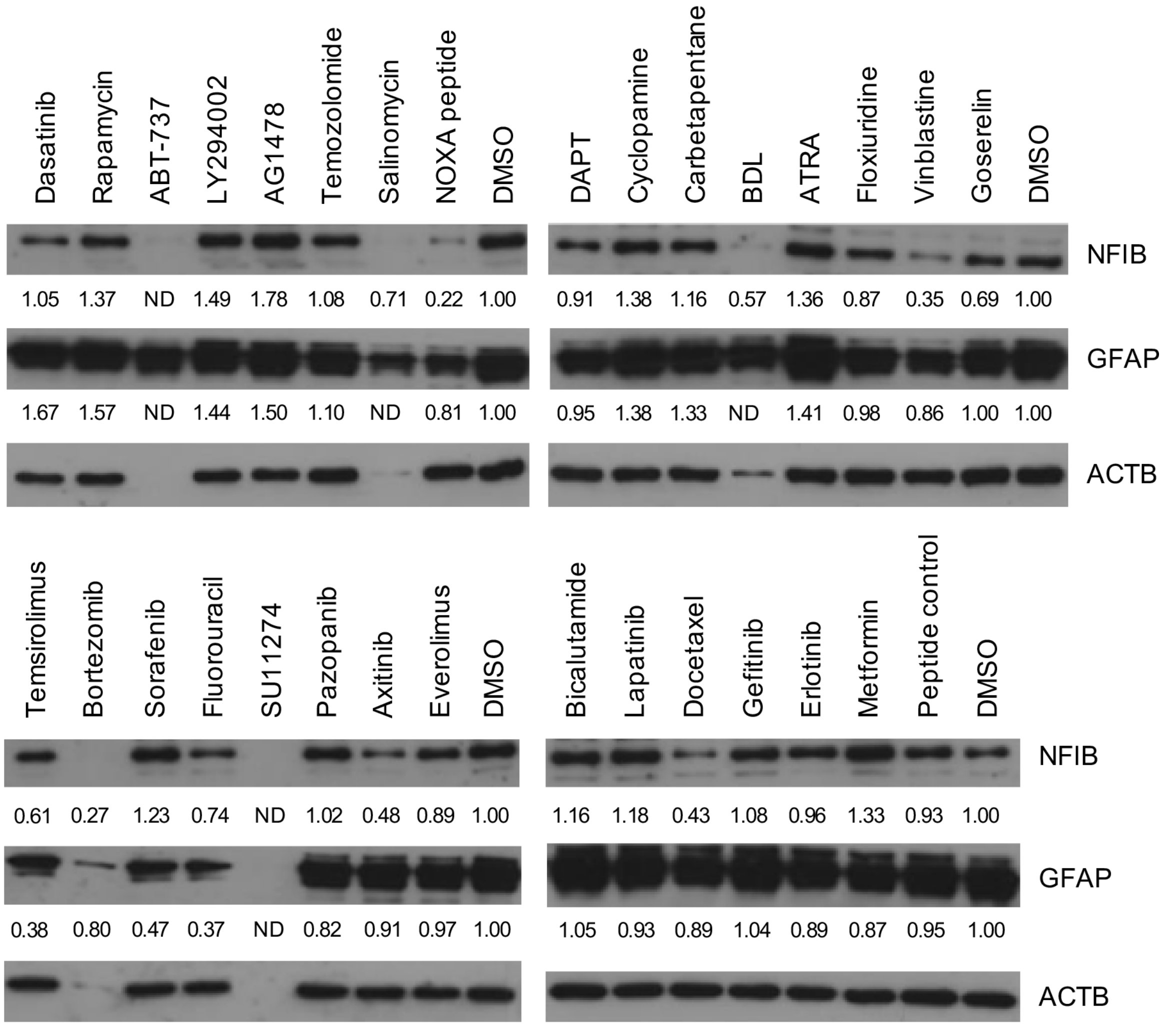

Supplementary Figure S2: Pilot drug screen for the induction of NFIB expression in the low-passage, unsubtyped GBM cell line Q1. 1.5×10^5 cells were cultured as adherent monolayers, in matrigel-coated 12-well plates in serum-free RHB-A medium supplemented with 20 ng/ml EGF and 10 ng/ml FGFb. Three hours after plating, cells were treated with 10 µM drug for 72 hours. Western blots were performed with 30 µg of total cell lysate and probed with antibodies against NFIB and the astrocytic differentiation marker GFAP. Numbers below the NFIB and GFAP panels represent the fold-change in western blot signal intensity, measured by densitometry, relative to DMSO-treated control cells and normalised to β-actin expression.
